# Genomic characterization of two *Staphylococcus epidermidis* bacteriophages with anti-biofilm potential

**DOI:** 10.1186/1471-2164-13-228

**Published:** 2012-06-08

**Authors:** Diana Gutiérrez, Beatriz Martínez, Ana Rodríguez, Pilar García

**Affiliations:** 1 DairySafe Group, Department of Technology and Biotechnology of Dairy Products, Instituto de Productos Lácteos de Asturias (IPLA-CSIC), Paseo Río Linares, s/n, 33300, Villaviciosa, Asturias, Spain

## Abstract

**Background:**

*Staphylococcus epidermidis* is a commensal bacterium but can colonize the hospital environment due to its ability to form biofilms favouring adhesion to host tissues, medical devices and increasing resistance to antibiotics. In this context, the use of phages to destroy biofilms is an interesting alternative.

**Results:**

The complete genomes of two *Staphylococcus epidermidis* bacteriophages, vB_SepiS-phiIPLA5 and vB_SepiS-phiIPLA7, have been analyzed. Their genomes are 43,581 bp and 42,123 bp, and contain 67 and 59 *orf*s. Bioinformatic analyses enabled the assignment of putative functions to 36 and 29 gene products, respectively, including DNA packaging and morphogenetic proteins, lysis components, and proteins necessary for DNA recombination, regulation, modification and replication. A point mutation in vB_SepiS-phiIPLA5 lysogeny control-associated genes explained its strictly lytic behaviour. Comparative analysis of phi-IPLA5 and phi-IPLA7 genome structure resembled those of *S. epidermidis* ϕPH15 and ϕCNPH82 phages. A mosaic structure of *S. epidermidis* prophage genomes was revealed by PCR analysis of three marker genes (integrase, major head protein and holin). Using these genes, high prevalence (73%) of phage DNA in a representative *S. epidermidis* strain collection consisting of 60 isolates from women with mastitis and healthy women was determined. Putative pectin lyase-like domains detected in virion-associated proteins of both phages could be involved in exopolysaccharide (EPS) depolymerization, as evidenced by both the presence of a clear halo surrounding the phage lysis zone and the phage-mediated biofilm degradation.

**Conclusions:**

*Staphylococcus epidermidis* bacteriophages, vB_SepiS-phiIPLA5 and vB_SepiS-phiIPLA7, have a mosaic structure similar to other widespread *S. epidermidis* prophages. Virions of these phages are provided of pectin lyase-like domains, which may be regarded as promising anti-biofilm tools.

## Background

*Staphylococcus epidermidis* is a common skin and mucous commensal of healthy humans, and can easily be transmitted to medical devices being a serious clinical problem and one of the major causes of nosocomial infections [1] as well as mastitis in lactating women [2]. In the animal health context *S. epidermidis* has also been recognized as one of the main etiological agents of ovine and bovine mastitis [3].

*S. epidermidis* is a key factor in the transmission of virulence factors and it is involved in balancing epithelial microbiota. In contrast to *S. aureus*, *S. epidermidis* does not encode many virulence factors, but it can colonize the hospital environment due to its ability to form biofilms favouring adhesion to host tissues, medical devices and increasing resistance to antibiotics [4]. In addition, the enormous flexibility of this bacterium continuously generates continuously novel phenotypic and genotypic variants. Hospital isolates are often characterised by the carriage of several staphylococcal chromosome cassettes (SCCmec), conferring methicillin resistance [5]. Moreover, nosocomial *S. epidermidis* strains typically harbour multiple copies of the insertion sequence element IS256 in their genomes, which contribute to genetic adaptation during infection [6]. Recently, the first *S. epidermidis* pathogenicity island (SePI), which encodes the staphylococcal enterotoxin SEC3 and SElL, has been described [7].

The widespread use of antibiotics in both humans and animals has led to the emergence of infectious bacteria resistant to a wide range of antimicrobials that greatly hinders their treatment. As a result of the search for complementary agents to antibiotics, phage therapy has resurfaced as means to prevent and treat infectious diseases. Phages have already been tested as anti-infectives in humans and animals [8], and phage-encoded lytic proteins may also be used to inhibit pathogenic bacteria [9]. In addition, the use of phages to destroy biofilms has gained much interest over the past years [10]. However, scarce information exists regarding the role of phages in eliminating *S. epidermidis* biofilms [11,12]. This is probably due to the limited number of phages infecting this species that have been characterized so far [13-15].

We have previously isolated and characterized three phages infecting *S. epidermidis* strains which belong to the *Siphoviridae* family (vB_SepiS-phiIPLA5, vB_SepiS-phiIPLA6, and vB_SepiS-phiIPLA7) [15]. Phage vB_SepiS-phiIPLA5 (hereafter phi-IPLA5) behaved as a virulent phage, probably derived from vB_SepiS-phiIPLA6, while vB_SepiS-phiIPLA7 (phi-IPLA7) was temperate. Both phages exhibited plaques surrounded by an increasing halo zone indicative of a polysaccharide depolymerase activity [16]. Moreover, in challenge assays phi-IPLA5 had lytic capability against *S. epidermidis*[15].

In the present work, the complete genome of phages phi-IPLA5 and phi-IPLA7 has been sequenced, annotated and compared with those previously described for staphylococcal phages. Genes encoding putative depolymerase activities were identified in these genomes. In addition, a representative *S. epidermidis* strain collection has been analyzed by using a multiplex PCR and the frequency of certain prophage groups determined. This study thus provides the basis for the evaluation of phages to control *S. epidermidis* strains.

## Results and discussion

Due to the renewed interest in phage therapy and the ability of phages to successfully combat infections in both animals and humans, the aim of this work was the genetic characterization of two new *S. epidermidis* phages (phi-IPLA5 and phi-IPLA7) to investigate their potential as antimicrobials and, more specifically, as anti-biofilm agents based on our previous observations of the presence of an increasing halo surrounding the lysis plaques, indicating a depolymerase activity [15].

### Genome overview of phi-IPLA5 and phi-IPLA7 phages

**Figure 1:**
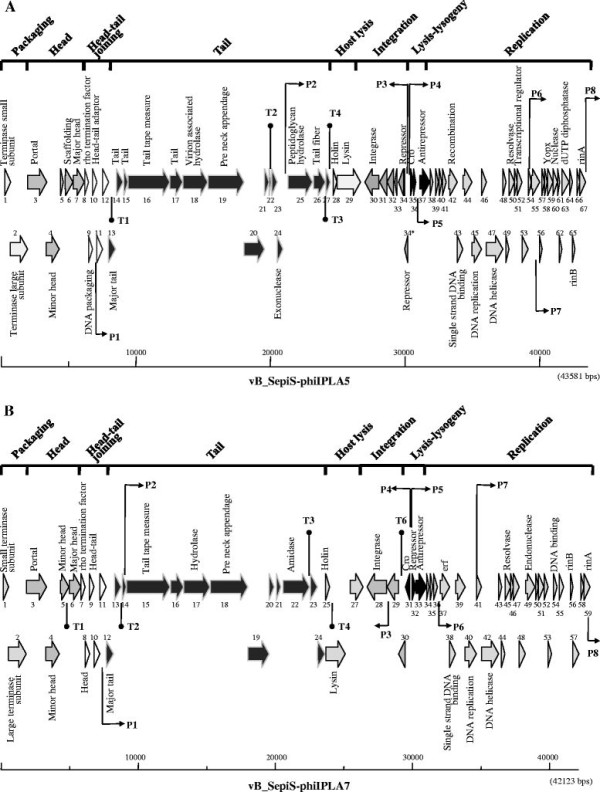
**Physical and genetic map of phages phi-IPLA5 (A) and phi-IPLA7 (B)**. The ORFs are sequentially numbered, indicated by arrows proportional to their lengths and pointing toward their direction of transcription. Some ORFs have been placed below for clarity. The functional modules are indicated on top of the scheme, and the names of several putatively or experimentally identified genes are shown. Putative promoter (P) and terminator (T) sequences are also indicated.

Both phages have a linear, double-stranded DNA genome consisting of 43,581 bp encoding 67 putative *orf*s in phi-IPLA5, while the phi-IPLA7 genome was 42,123 bp and 59 putative *orf*s were identified (Additional file 1: Table S1 and Additional file 2: Table S2; Figure 1A and B)**.** The G + C content of phi-IPLA5 and phi-IPLA7 was 34.7%, which is slightly higher than that of *S. epidermidis* strains (32%) [17]. A BLASTN search revealed that nucleotide sequence of phi-IPLA5 and phi-IPLA7 shared a high degree of similarity with the other two *S. epidermidis* phages phiPH15 and phiCNPH82 (64% and 65%, for phi-IPLA5, and 81% and 67% for phi-IPLA7, respectively) [14]. Bioinformatic analysis revealed a similar organization of the two phages in five functional modules (packaging, structure/morphogenesis, host lysis, lysogeny and replication/regulation) that perfectly fits the general structure of most double-stranded DNA bacteriophages [18]. Several putative promoters and terminators in phi-IPLA5 and in phi-IPLA7 were found by searching for the *S. aureus* σ^70^-dependent promoter consensus motif (Figure 1A and B, Additional file 3: Table S3). The deduced promoter positions are consistent with a modular arrangement and imply that there is modular control of gene expression. The putative transcription pattern of both phages reveals two main groups of *orfs* running divergently and one of them includes the lysogenic cassette.

The amino acid (aa) sequences of the predicted *orfs* were searched for similarities to sequences from the available databases (Additional file 1: Table S1 and Additional file 2: Table S2). Significant matches were obtained for 36 *orf*s from phi-IPLA5 and 29 *orf*s from phi-IPLA7 and biological functions were assigned. No tRNA genes were found. No virulence genes were clearly identified.

In the DNA packaging module, the putative large terminase subunit of phi-IPLA5 and phi-IPLA7 showed homology (59%) with those belonging to the *pac*-type phages such as *Bacillus subtilis* phage SPP1. It has been suggested that different functional classes of phage-encoded terminases can be predicted from their amino acid sequence [19]. Confirmation of this result was obtained by restriction analysis of phi-IPLA5 and phi-IPLA7 DNAs with the endonuclease *Xba*I. The phage genomes both have two cut sites but produced two single bands on agarose gels (data not shown), which suggests that both phi-IPLA5 and phi-IPLA7 genomes were circularly permuted.

In the structural module, the predicted major head and major tail proteins had a molecular mass consisting with previous protein analysis of virion particles, which showed a major polypeptide (34 kDa) in phage phi-IPLA5 and two main proteins (27.5 and 34 kDa) in phi-IPLA7, respectively [15]. Putative virion-associate hydrolases (phi-IPLA5 gp18 and phi-IPLA7 gp17) with an aminoterminal endopeptidase tail domain and a SGNH hydrolase domain related with lipases and esterases, were identified. Finally, pre neck appendage proteins (phi-IPLA5 gp19 and phi-IPLA7 gp18) with pectin-lyase like domains weer identified that could also be involved in the extracellular material degradation.

Phage phi-IPLA5, although strictly lytic, encoded a deficient lysis-lysogeny module. The phi-IPLA5 gp34 and phi-IPLA5 gp34* proteins shared extended similarity with repressors of the CI type. A one-base replacement that shifted a TAC codon to the stop codon TAA was mapped at 29941 nt resulting in a second *orf* (orf34*). No RBS upstream of orf34* could be detected. The presence of a truncated CI repressor in phi-IPLA5 would explain why the phage was unable to lysogenize [15]. In the *cI-cro* intergenic regions of both phages, two adjacent and outward-facing putative promoters for *cro* and repressor genes were identified (Figure 1A and B, Additional file 3: Table S3). Additionally, two 7-bp direct repeats overlapping the two putative promoters in phi-IPLA7 were recognized (data not shown). These sequences might be putative operators for the binding of CI repressor which have been reported as regulators in the lysogeny module gene expression [20].

In the replication module, both phages contained DNA replication proteins (phi-IPLA5 gp45 and phi-IPLA7 gp40) with DnaB domains which are essential in replication initiation, as well as DnaD domains which are a component of the primosome. phi-IPLA5 gp50 and phi-IPLA7 gp45 displayed homology to a Holliday junction resolvase (RusA) [21]. phi-IPLA5 gp59 had homology with Yopx proteins, an uncharacterized, well-conserved family of proteins found in bacteriophage and prophage regions of Gram-positive bacteria. A putative dUTPase gene gp63 was predicted in phi-IPLA5 genome which is highly conserved in several staphylococcal and lactococcal phages [22,23]. Gp65 and gp67 from phi-IPLA5 and gp56 and gp59 from phi-IPLA7 displayed similarity to the RinA and RinB family of transcriptional regulators. Recently, the RinA family proteins have been showed as activators required for transcription of the late operon in temperate staphylococcal phages [24].

### Comparative genomics of phi-IPLA5 and phi-IPLA7 phages

**Figure 2:**
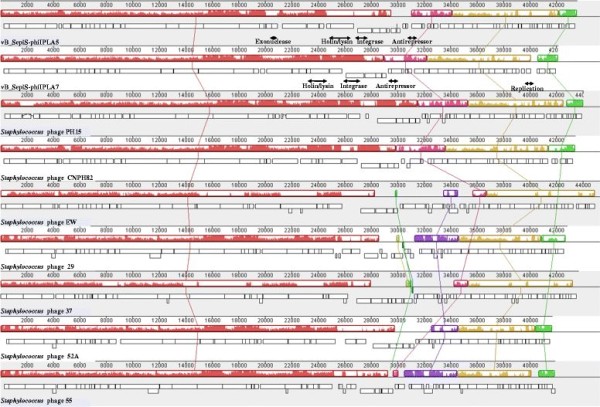
**Alignment of the genome of the two *S. epidermidis *phages phi-IPLA5 and phi-IPLA7 with those of other *Staphylococcus *phages using the Mauve software**. Each block represents a region of the genome sequence that aligned and is homologous to part of another genome. Regions outside blocks lack detectable homology among the input genomes. Inside each block nucleotide sequence similarity is indicated by the height of the colored bars, while regions that are dissimilar are in white. Lines connecting blocks are indicative of homologous regions.

BLASTN database searches with the complete genome sequence of phi-IPLA5 and phi-IPLA7 revealed similarity at the nucleotide level with the two previously described *S. epidermidis* phages PH15 and CNPH82 and with *S. aureus* phages such as phiEW, phi29, phi37, phi52A and phi55. Comparison of these phages using Mauve software revealed gene synteny among the four *S. epidermidis* phages. Four homology blocks mostly matched the morphogenesis and integration, the lysis-lysogeny, replication, and regulation modules, (Figure 2). Extensive similarity in the head and tail morphogenesis modules was observed among the *S. epidermidis* phages genomes and other *Staphylococcus* phages (Figure 2; Additional file 1: Table S1 and Additional file 2: Table S2). In fact, *S. epidermidis* phages PH15 and CNPH82 have been shown to be highly similar to class II clade C of *S. aureus* phages [14]. An exception was observed in the putative exonuclease encoding gene (*orf*24) from phi-IPLA5, which was not found in phi-IPLA7 or in other staphylococcal phages. The phi-IPLA5 lysis region seemed to be rather unique, specifically in the holin encoding gene (*orf*28), to which no similarity was detected within other *S. epidermidis* phages. The holin phi-IPLA5 gp28 protein showed similarity (50%) to the holin from *S. aureus* phage 29 while the holin phi-IPLA7 gp25 protein was identical to those in phages PH15 and CNPH82. Similarly, the phi-IPLA5 lysin encoding gene (*orf*29) did not have any matching gene in other phages. Moreover, the lysin phi-IPLA5 gp29 protein belongs to the amidase_2 family while those of phi.IPLA7 (gp26) and the lysins from *S. epidermidis* phages PH15 and CNPH82 were highly similar (99%) and belong to the amidase_3 family. A similar pattern was observed in the region surrounding the integrase gene. The putative integrase gene (*orf*30) of phi-IPLA5 differed from that in phi-IPLA7, and the corresponding protein (gp30) was 70% similar to that of *S. aureus* phi96, while the integrase phi-IPLA7 protein (gp28) resembled that of *S. epidermidis* PH15 and CNPH82. The phage integrases from the newly isolated phages belonged to different families (Additional file 1: Table S1 and Additional file 2: Table S2).

Finally, no similarity with other phages was detected for the antirepressor encoding gene (*orf*33) found in the phi-IPLA7 lysis-lysogeny region, Based on these data, *S. epidermidis* PH15 and CNPH82 and *S. aureus* phages phiEW, phi29, phi37, phi52A and phi55 are the closest relatives to phi-IPLA5 and phi-IPLA7. The extensive similarity between these phages is mainly in the morphogenetic region as previously reported on other *Siphoviridae* phages [25]. The observed homology in other regions or modules supports the modular theory of phage evolution [26].

### Incidence and typing of prophages in *S. epidermidis* strains

Previous studies have shown that prophages integrated in the bacterial chromosomes are the most widespread mobile genetic elements in *S. aureus* strains, which tipically carry between one and four prophages [27]. However, the prophage content in *S. epidermidis* has not been determined to date. To approach this, the integrase (*int*), holin (*hol*) and major head protein (*mhp*) genes were selected as marker modules as previously described in by [28] who studied genome mosaicism in prophages of *S. aureus*. Based on the genome wide comparison among phages and further *in silico* analysis of the integrase genes from the four *S. epidermidis* phages (phi-IPLA5, phi-IPLA7, PH15 and CNPH82), two groups were identified: int1 comprised by the highly similar *int* genes from phi-IPLA7, PH15 (95%) and CNPH82 (99%) and int2 composed by *int* phi-IPLA5 to which no similarities were found among them. Likewise, *S. epidermidis* phage holin genes defined two groups: hol1 group comprised by *hol* phi-IPLA7, PH15 (91%) and CNPH82 (100%) and hol2 group that included *hol* phi-IPLA5. Finally, the phi-IPLA7 *mhp* gene was very similar to phi-IPLA5 (97%) and PH15 (95%) but no counterpart was identified in CNPH82. Based on amino acid sequence homology this protein belongs to the phage-capsid superfamily. The presence or absence of this gene generated the mhp1 and mhp2 groups, respectively.

The presence of prophages in our *S. epidermidis* collection containing 60 isolates from women breast milk was investigated by a multiplex PCR to amplify the above mentioned genes (*int*, *hol* and *mhp*). Prophages of the group mhp1 were the most frequent (30%) but none of the other targeted markers were amplified in these strains (Additional file 4: Table S4). About 10% of the isolates were included in the mhp2 group and were mostly associated to the int2 marker. We also observed that some phage groups were completely absent and others were less frequent. There were also some strains in which no amplification was observed pointing to the absence of prophages or the presence of other genes not detected by our PCR approach. It is remarkable that, besides the 10 *S. aureus* bacteriophage integrase gene classes analysed previously [28], many other types of modules containing lysogenic functions, DNA replication, packaging, tail appendices and host lysis were described in *S. aureus* phages, revealing the high diversity and the mosaic structure of prophages in this species [22].

Previous studies suggested that the integrase type group in *S. aureus* strains is closely linked to the virulence gene content of prophages and might therefore convey information about their pathogenic potential [28]. We found that the largest group of *S. epidermidis* prophages belongs to int2 group, which was mostly found in mastitic strains. On the other hand, prevalence of prophages, as defined by the positive amplification of at least one marker gene, was higher (82% vs 59%) in strains producing mastitis. This result would support the hypothesis that prophages are directly involved in virulence [29]. However, no virulence factors have been described to date in *S. epidermidis* phages.

In our previous work, the yield of mitomycin C inducible prophages in *S. epidermidis* was rather low (3%) and we hypothesized that it could be due to the lack of appropriately sensitive host strains to detect them [15]. In view of the new PCR results, phage DNA sequences were present in 73% of the analyzed *S. epidermidis* strains. Although amplification of at least one of the markers does not necessarily mean that the bacterial strains are lysogens (i.e. marker genes in defective prophages), our results support the notion that lysogeny in *S. epidermidis* may be higher than anticipated and likely more frequent among clinical strains, as noted previously in *S. aureus* strains isolated from diverse clinical samples [28].

### Virion proteins with a putative hydrolytic activity of extracellular components

**Figure 3:**
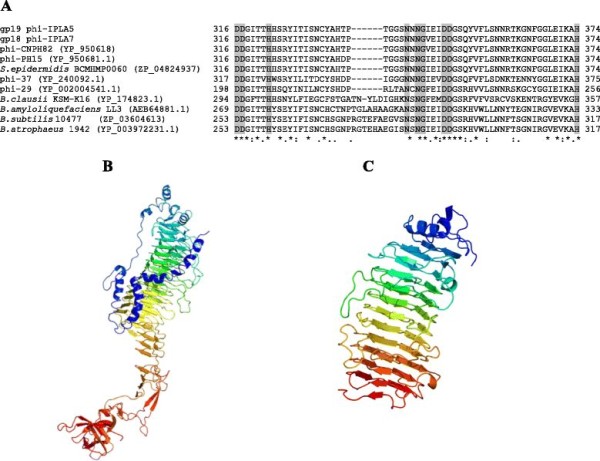
**Aminoacid sequence analysis of the virion-associated protein gp18 from phi-IPLA7.A)** ClustalW alignment of the predicted binding site. Positions with a single, fully conserved residue are marked with an asterisk; the colon marks the conserved residues between groups of strongly similar properties, and the period marks the residues weakly conserved between groups based on the Gonnet PAM 250 matrix, score <0,5. Highlighted are those residues involved in ligand association. **B)** Predicted 3D structure of phi-IPLA7 gp18. **C)** Predicted 3D structure of the domain pectin lyase like (D1) (amino acids 316- 374).

A pectin lyase-like domain (aminoacids 117 to 539) was identified in the pre-neck appendage protein of both phages (Additional file 1: Table S1 and Additional file 2: Table S2). Recently, it has been demonstrated that some tail spike proteins similar to pectin lyases have exopolysaccharide (EPS) degrading activity [30]. Similar proteins to phi-IPLA5 gp19 and phi-IPLA7 gp18 were identified by BLASTp search in PH15 and CNPH82 phages, *S. aureus* phage 37, *Bacillus* phage phi29 and prophages in several *Bacillus* species (Figure 3A). Surprisingly, most of the phi-IPLA5 gp19 and phi-IPLA7 gp18 related proteins belonged to *Bacillus* sp and not to other staphylococcal species. It is remarkable that all the proteins analyzed were highly conserved at the predicted binding site (amino acids 316-374) and the residues responsible for the interaction with the ligand were shared by the different phages (Figure 3A). To support the predicted catalytic domains, the software Phyre^2^ was used to predict the tridimensional structure (3day) of the protein phi-IPLA7 gp18 to which, the primary structure of the counterpart protein from phi-IPLA5 was 98% identical. To model phi-IPLA7 gp18 (Figure 3B), the crystal structure of the bacteriophage phi29 gp12 neck protein from *Bacillus subtilis*[31] was used. 82% of phi-IPLA7 gp18 amino acids were predicted at a confidence level of 90%. The pectin lyase-like domain (D1) (Figure 3C) displayed a right-handed continuous twelve helix repetition, where each coil of the helix featured three beta-strands and three turn regions. Proteins containing these repeats most often are enzymes with polysaccharide substrates, and it was demonstrated that this topology is shared by several proteins, with the variation in the number of coils, including bacterial pectate lyases, fungal and bacterial galacturonases and phage tail spikes [32]. On the other hand, no amino acid sequence homology was detected between phi-IPLA7 gp18 and *Pseudomonas putida* ϕ15 tail spike protein, although this also showed right handed beta helical folds identified in carbohydrate depolymerizing enzymes [30].

### Biofilm degradation by phi-IPLA5 and phi-IPLA7 phages

**Figure 4:**
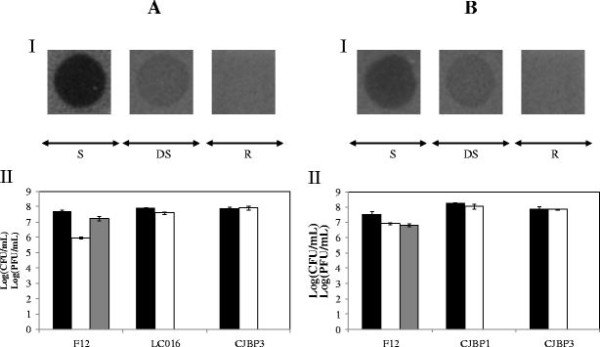
**Effect of phages A) phi-IPLA5 and B) phi-IPLA7 on a lawn of *S. epidermidis*.I)** Morphology of phage lytic zone when dropped on different *S. epidermidis* strains. (S) Sensitive strain; (R) resistant strain; (DS) drop-sensitive strain. **II)** Bacteria viable number in drop-zone (white), bacteria lawn (black) and phage titre (grey) of phages. Each value correspond with the mean of five different experiments, the standard error is represented by bars.

Three lines of evidence were pointing to a putative anti-biofilm activity by the newly isolated *S. epidermidis* phages phi-IPLA5 and phi-IPLA7. First, as previously reported [15], a halo surrounding the clear lysis plaques could be observed which increased with time. These halos are regarded as an indicator for the presence of phage-associated EPS depolymerases [16,30]. Secondly, a clear zone was previously observed when phages phi-IPLA5 and phi-IPLA7 were dropped on some bacterial strains (drop-sensitive strains). These strains were resistant to infection meaning that no plaques were observed when the phage stock was diluted and plated on these strains [15]. Third, based on sequence comparison and 3D predictions, two candidate proteins were identified as putative EPS degrading enzymes. To investigate if these phages were involved in biofilm degradation, cell counts and phage titre of the clear zone where the phage suspension is dropped, and into the host cell lawn were carried out. As expected, lower bacterial counts were found when the phages were dropped onto the sensitive strain *S. epidermidis* F12 while viable counts of the resistant *S. epidermidis* CJBP3 remained unaltered (Figure 4). Interestingly, the clear zone generated on the drop-sensitive strains revealed similar bacterial counts to the bacterial lawn despite its distinct visual appearance. Moreover, *S. epidermidis* F12 clearly supported phage propagation, while no phages were detected when sampling the drop zone of the resistant and drop sensitive strains (Figure 4). Overall, these results supports the presence of phage depolymerase activity, although we can not totally discard our previous hypothesis that ascribed the clear zone on drop-sensitive strains to “lysis from without” [15].

**Figure 5:**
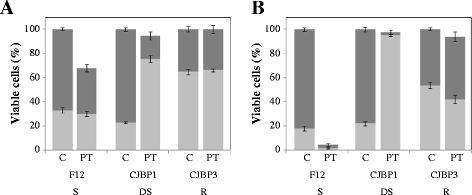
**Killing of *S. epidermidis *cells forming a biofilm on microtiter wells by phage phi-IPLA7 after incubation for 1 h (A) and 3 h (B).** Results are depicted as the percentage of attached cells (*dark gray square*) and planktonic cells (*light gray square*) detected in control biofilms treated with SM buffer **(C)** and in biofilms treated with phage phi-IPLA7 (PT). Each value corresponds with the mean of five different experiments and the standard error is represented by bars

In order to confirm the ability of the phages to mediate biofilm degradation, the three types of strains were grown in microtiter plates for 7 days for biofilm formation and subsequently challenged with phage phi-IPLA7. Viability of the sensitive *S. epidermidis* F12 in both planktonic and biofilm cells was readily reduced even after 1 h in the presence of the phage, and only 5% of the total counts survived (ANOVA; p < 0.05) after 3 h (Figure 5). In the case of the drop-sensitive *S. epidermidis* CJBP1, total cell numbers remained unchanged (ANOVA; p > 0.05) at both 1 h and 3 h. However, a dramatic shift towards the planktonic state was observed in the presence of the phage and, after 3 h, and just 2% of the cells remained attached (Figure 5B). These data support the ability of phage phi-IPLA7 to degrade the extracellular material of the biofilm, releasing the attached cells. On the contrary, neither the viability nor the biofilm formed by the resistant strain *S. epidermidis* CJBP3 was affected by phage phi-IPLA7 (ANOVA; p > 0.05). A different composition in the extracellular biofilm material could explain this result. Studies on staphylococcal biofilm development suggest that the extracellular matrix consists of proteins, DNA, and/or polysaccharide (PIA) [1]. Recently, it has become evident that some strains are not reliant on PIA for biofilm formation [33].

## Conclusions

In this work, we have presented a detailed genomic and molecular characterization of two new *S. epidermidis* phages, phi-IPLA5 and phi-IPLA7. Based on this, a multiplex PCR was designed that revealed a high prevalence of prophage and/or defective prophages within *S. epidermidis* strains. Genome mining detected the presence of virion-associated proteins with a putative EPS depolymerase activity. To our knowledge, this is the first time that degradation of extracellular material has been ascribed to *S. epidermidis* phages. Further studies to confirm the role of these proteins in phage-induced biofim destructuring are in progress with the hope to set the foundation of new anti-biofilm strategies.

## Methods

### Bacterial growth conditions and phages propagation

*S. epidermidis* F12 was used as the host strain for phages phi-IPLA5 and phi-IPLA7 [15]. A total of sixty *S. epidermidis* strains isolated from women’s breast milk [2] were used in lysogeny analysis (Additional file 5: Figure S1). Staphylococcal cells were routinely cultured in TSB broth (Triptona Soy Broth, Scharlau, Barcelona, Spain) at 37°C with shaking or in TSB plates containing 2% (w/v) bacteriological agar (TSA). Bacteriophages phi-IPLA5 and phi-IPLA7 were propagated as described previously [15].

### Phage genome sequencing and analysis

To prepare bacterial DNA-free samples for sequence analysis, the purified phages were treated with DNaseI bovine pancreas (Sigma, Madrid, Spain) and the phage DNA was extracted as previously described [34]. The genome sequence of phages phi-IPLA5 and phi-IPLA7 was generated by ultra-high throughput GS FLX sequencing with 20-fold redundancy on average. *Orf*s were predicted with Clone Manager 7 version 7.10 software in all reading frames with a threshold of 40 codons. BLASTX and BLASTP ( http://www.ncbi.nlm.nih.gov/blast/Blast.cgi) were used to search for homologous proteins. Structural predictions and motif searches were performed with InterProScan ( http://www.ebi.ac.uk/InterProScan/). The search for putative tRNA encoding genes was performed with tRNAscan-SE 1.21 ( http://selab.janelia.org/tRNAscan-SE/). σ^70^ promoter sequences were identified using PPP (Prokaryotic Promoter Prediction at http://bioinformatics.biol.rug.nl/websoftware/ppp/ppp_start.php). The rho-independent terminators were identified using the Trans Term program ( http://nbc3.biologie.uni-kl.de) and energy was calculated by the mfold web server ( http://mfold.rna.albany.edu). Genomic comparisons at the nucleotide level were made with Mauve software, using a progressive alignment with default settings ( http://gel.ahabs.wisc.edu/mauve/). 3 day structure prediction of proteins was made by using the bioinformatic software Phyre^2^ ( http://gel.ahabs.wisc.edu/mauve/download.php). To predict the binding site of the ligands, the software 3Dligandsite was used ( http://www.sbg.bio.ic.ac.uk/3dligandsite/).

### Accession numbers

The sequences of phi-IPLA5 and phi-IPLA7 have been deposited in the GenBank under accession numbers JN192400 and JN192401, respectively.

### Lysogeny and prophage typing

The presence of resident prophages was screened by multiplex PCR using the primers specified in Additional file 5: Figure S1. Total DNA extraction was carried out from *S. epidermidis* strains by using *“GenElute*™*Bacterial Genomic DNA Kit”* (Sigma-Aldrich, Madrid, Spain). PCR reactions were performed using the kit *‘PureTaq Ready-To-Go*™*PCR Beads’* (GE Healthcare, Munich, Germany). As positive control, pure phage DNA from phi-IPLA5 and phi-IPLA7 was used. Gel images were processed using the software Quantity One software (BioRad Laboratories, Hercules, CA). The similarity matrix was calculated on the basis of the simple matching coefficient, and its corresponding dendrogram was deduced using the unweighted pair group method with arithmetic averages.

### Microbiological analysis of phage lysis zones and biofilms

To visualize the lysis area formation, 5 μl of a 10^8^ PFU/ml phage suspension was dropped on a *S. epidermidis* bacterial lawn and incubated at 37°C. For comparison between the lysis zone and the bacterium lawn zone, an area with the same volume was removed from each zone, suspended in 200 μl of SM buffer (20 mM Tris–HCl, 10 mM MgSO_4_, 10 mM CaCl_2_, 100 mM NaCl, pH 7.5) and vigorously vortexed. Phage titre was determined using the double layer agar method, while bacterial count was determined by plating serial dilutions on TSB agar. To determine the potential of phages to degrade biofilms, *S. epidermidis* o/n cultures were diluted in fresh TSB to 10^6^ CFU/ml, poured into a 96 microwell plate (Thermo Scientific, Madrid, Spain) and incubated during 7 day at 37°C. Wells were washed twice with SM buffer and either 220 μl of a phage stock (10^8^ PFU/ml) or 220 μl of SM buffer were added for test and control purposes, respectively. Plates were incubated for 1 h and 3 h and then supernatants and adhered cells were collected and plated for bacteria counting. The results were represented as the viable cells percentage respect the total cell number in the control wells without phage treatment (cells in the supernatant + cells adhered to the well).

Statistical analysis was performed by one-way analysis of variance (ANOVA) followed by the Bonferroni multi-comparison test. Statistical significance was considered at p < 0.05.

## Competing interests

The authors declare that they have no competing interests.

## Authors’ contributions

DG and PG performed bioinformatics analyses of nucleotide and protein sequences. PG, BM and AR designed the study, obtained funding and wrote the manuscript. All authors read and approved the final manuscript.

## Supplementary Material

Additional file 1 **Table S1.** Features of bacteriophage phi-IPLA5 *orf*s, gene products (gp) and functional assignments. Click here for file

Additional file 2 **Table S2.** Features of bacteriophage phi-IPLA7 *orf*s, gene products (gp) and functional assignments. Click here for file

Additional file 3 **Table S3.** Putative promoters and terminators sequences of phi-IPLA5 and phi-IPLA7. -10 and -35 boxes are underlined. Nucleotide positions and presence of the TG dinucleotide were also indicated. At terminator sequences nucleotides in the stem-loop structure are underlined. Click here for file

Additional file 4 **Table S4.** Primers used for multiplex PCR reactions. Click here for file

Additional file 5 **Figure S1.** Multiplex PCR detecting the integrase, holin and major head protein genes in *S. epidermidis* strains. (H) Strains isolated from healthy woman, (M) strains isolated from mastitic women. Absence and presence of a specific gene is represented by white and grey boxes, respectively. Click here for file

## References

[B1] Otto M Staphylococcus epidermidis the “accidental” pathogen Nat Rev 2009 7 555 567 10.1038/nrmicro2182 PMC2807625 19609257

[B2] Delgado S Arroyo R Jiménez E Marín ML Del Campo R Fernández L Rodríguez JM Staphylococcus epidermidis strains isolated from breast milk of women suffering infectious mastitis: potential virulence traits and resistance to antibiotics BMC Microbiol 2009 9 82 10.1186/1471-2180-9-82 19422689 PMC2685400

[B3] Oliveira M Nunes SF Carneiro C Bexiga R Bernardo F Vilela CL Time course of biofilm formation by Staphylococcus aureus and Staphylococcus epidermidis mastitis isolates Vet Microbiol 2007 124 187 191 10.1016/j.vetmic.2007.04.016 17509779

[B4] Jabbouri S Sadovskaya I Characteristics of the biofilm matrix and its role as a possible target for the detection and eradication of Staphylococcus epidermidis associated with medical implant infections FEMS Immunol Med Microbiol 2010 59 280 291 20528930 10.1111/j.1574-695X.2010.00695.x

[B5] Schoenfelder SM Lange C Eckart M Hennig S Kozytska S Ziebuhr W Success through diversity - how Staphylococcus epidermidis establishes as a nosocomial pathogen Int J Med Microbiol 2010 300 380 386 10.1016/j.ijmm.2010.04.011 20451447

[B6] Kozitskaya S Cho SH Dietrich K Marre R Naber K Ziebuhr W The bacterial insertion sequence element IS256 occurs preferentially in nosocomial Staphylococcus epidermidis isolates: association with biofilm formation and resistance to aminoglycosides Infect Immun 2004 72 1210 1215 10.1128/IAI.72.2.1210-1215.2004 14742578 PMC321601

[B7] Madhusoodanan J Seo KS Remortel B Park JY Hwang SY Fox LK Park YH Deobald CF Wang D Liu S Daugherty SC Gill AL Bohach GA Gill SR An enterotoxin-bearing pathogenicity island in Staphylococcus epidermidis J Bacteriol 2011 193 1854 1862 10.1128/JB.00162-10 21317317 PMC3133018

[B8] Kutateladze M Adamia R Bacteriophages as potential new therapeutics to replace or supplement antibiotics Trends Biotechnol 2010 28 591 595 10.1016/j.tibtech.2010.08.001 20810181

[B9] Fischetti VA Bacteriophage endolysins: a novel anti-infective to control Gram-positive pathogens Int J Med Microbiol 2010 300 357 362 10.1016/j.ijmm.2010.04.002 20452280 PMC3666336

[B10] Donlan RM Preventing biofilms of clinically relevant organisms using bacteriophage Trends Microbiol 2009 17 66 72 10.1016/j.tim.2008.11.002 19162482

[B11] Cerca N Oliveira R Azeredo J Susceptibility of Staphylococcus epidermidis planktonic cells and biofilms to the lytic action of Staphylococcus bacteriophage K Lett Appl Microbiol 2007 45 313 317 10.1111/j.1472-765X.2007.02190.x 17718845

[B12] Curtin JJ Donlan RM Using bacteriophages to reduce formation of catheter-associated biofilms by Staphylococcus epidermidis Antimicrob Agents Chemother 2006 50 1268 1275 10.1128/AAC.50.4.1268-1275.2006 16569839 PMC1426991

[B13] Aswani V Tremblay DM Moineau S Shukla SK Staphylococcus epidermidis bacteriophages from the anterior nares of humans Appl Environ Microbiol 2011 77 7853 7855 10.1128/AEM.05367-11 21926216 PMC3209164

[B14] Daniel A Bonnen PE Fischetti VA First complete genome sequence of two Staphylococcus epidermidis bacteriophages J Bacteriol 2007 189 2086 2100 10.1128/JB.01637-06 17172342 PMC1855768

[B15] Gutiérrez D Martínez B Rodríguez A García P Isolation and characterization of bacteriophages infecting Staphylococcus epidermidis Curr Microbiol 2010 61 601 608 10.1007/s00284-010-9659-5 20449591

[B16] Hughes KA Sutherland IW Jones MV Biofilm susceptibility to bacteriophage attack: the role of phage-borne polysaccharide depolymerase Microbiology 1998 144 3039 3047 10.1099/00221287-144-11-3039 9846739

[B17] Cates S NCBI: National Center for Biotechnology Information 2006 http://cnx.org/content/m11789/1.3/

[B18] Brüssow H Desiere F Comparative phage genomics and the evolution of Siphoviridae: insights from dairy phages Mol Microbiol 2001 39 213 222 10.1046/j.1365-2958.2001.02228.x 11136444

[B19] Casjens SR Gilcrease EB Winn-Stapley DA Schicklmaier P Schmieger H Pedulla ML Ford ME Houtz JM Hatfull GF Hendrix RW The generalized transducing Salmonella bacteriophage ES18: complete genome sequence and DNA packaging strategy J Bacteriol 2005 187 1091 1104 10.1128/JB.187.3.1091-1104.2005 15659686 PMC545730

[B20] García P Ladero V Alonso JC Suárez JE Cooperative interaction of CI protein regulates lysogeny of Lactobacillus casei by bacteriophage A2 J Virol 1999 73 3920 3929 10196287 10.1128/jvi.73.5.3920-3929.1999 PMC104170

[B21] Mahdi AA Sharples GJ Mandal TN Lloyd RG Holliday junction resolvases encoded by homologous rusA genes in Escherichia coli K-12 and phage 82 J Mol Biol 1996 257 561 573 10.1006/jmbi.1996.0185 8648624

[B22] Kahánková J Pantůček R Goerke C Růžičková V Holochová P Doškař J Multilocus PCR typing strategy for differentiation of Staphylococcus aureus siphoviruses reflecting their modular genome structure Environ Microbiol 2010 12 2527 2538 10.1111/j.1462-2920.2010.02226.x 20406289

[B23] Labrie S Moineau S Complete genomic sequence of bacteriophage ul36: demonstration of phage heterogeneity within the P335 quasi-species of lactococcal phages Virol 2002 296 308 320 10.1006/viro.2002.1401 12069529

[B24] Ferrer MD Quiles-Puchalt N Harwich MD Tormo-Más MA Campoy S Barbé J Lasa I Novick RP Christie GE Penadés JR RinA controls phage-mediated packaging and transfer of virulence genes in Gram-positive bacteria Nucleic Acids Res 2011 39 5866 5878 10.1093/nar/gkr158 21450808 PMC3152322

[B25] Proux C van Sinderen D Suarez J García P Ladero V Fitzgerald GF Desiere F Brüssow H The dilemma of phage taxonomy illustrated by comparative genomics of Sfi21-like Siphoviridae in lactic acid bacteria J Bacteriol 2002 184 6026 6036 10.1128/JB.184.21.6026-6036.2002 12374837 PMC135392

[B26] Brüssow H Canchaya C Hardt WD Phages and the evolution of bacterial pathogens: from genomic rearrangements to lysogenic conversion Microbiol Mol Biol Rev 2004 68 560 602 10.1128/MMBR.68.3.560-602.2004 15353570 PMC515249

[B27] Pantůček R Doskar J Růzicková V Kaspárek P Orácová E Kvardová V Rosypal S Identification of bacteriophage types and their carriage in Staphylococcus aureus Arch Virol 2004 149 1689 1703 10.1007/s00705-004-0335-6 15593413

[B28] Goerke C Pantůček R Holtfreter S Schulte B Zink M Grumann D Bröker BM Doskar J Wolz C Diversity of prophages in dominant Staphylococcus aureus clonal lineages J Bacteriol 2009 191 3462 3468 10.1128/JB.01804-08 19329640 PMC2681900

[B29] Boyd EF Brüssow H Common themes among bacteriophage-encoded virulence factors and diversity among the bacteriophages involved Trends Microbiol 2002 10 521 529 10.1016/S0966-842X(02)02459-9 12419617

[B30] Cornelissen A Ceyssens PJ T’Syen J Van Praet H Noben JP Shaburova OV Krylov VN Volckaert G Lavigne R The T7-related Pseudomonas putida phage ϕ15 displays virion-associated biofilm degradation properties PLoS One 2011 6 e18597 10.1371/journal.pone.0018597 21526174 PMC3079711

[B31] Xiang Y Leiman PG Li L Grimes S Anderson DL Rossmann MG Crystallographic insights into the autocatalytic assembly mechanism of a bacteriophage tail spike Mol Cell 2009 34 375 386 10.1016/j.molcel.2009.04.009 19450535 PMC2692858

[B32] Jenkins J Mayans O Pickersgill R Structure and evolution of parallel beta-helix proteins J Struct Biol 1998 122 236 246 10.1006/jsbi.1998.3985 9724625

[B33] Tormo MA Knecht E Götz F Lasa I Penadés JR Bap-dependent biofilm formation by pathogenic species of Staphylococcus: evidence of horizontal gene transfer? Microbiology 2005 151 2465 2475 10.1099/mic.0.27865-0 16000737

[B34] Sambrook J Fritsch EF Maniatis T Molecular cloning: a laboratory manual 1989 2 Cold Spring Harbor, NY,Cold Spring Harbor Laboratory

