# Supplementary material for: Genomic characterization of two Staphylococcus epidermidis bacteriophages with anti-biofilm potential
Source: BMC Genomics. 2012 Jun 8;13:228. doi: 10.1186/1471-2164-13-228 (PMC3505474; doi:10.1186/1471-2164-13-228)
Supplement: Additional file 1 — Table S1. Features of bacteriophage phi-IPLA5 orfs, gene products (gp) and functional assignments. [file 1471-2164-13-228-S1.doc]

**Table 1.** Features of bacteriophage phi-IPLA5 *orf*s, gene products (gp) and functional assignments.

| ***orf*** | **From** | **To** | **Length** | **aa** | **kDa (pI)** | **Predictive Function** | **Closes hit (E value)** | **% aa identity / % similarity** | **Accesion no.** | **Predicted domain (E value)** |
| --- | --- | --- | --- | --- | --- | --- | --- | --- | --- | --- |
| **1** | 242 | 676 | 435 | 144 | 16.5 (4.98) | Terminase small subunit | *S. epidermidis* M23864 (4e-77) | 99% (100%) | ZP_06614648.1 | PF03592Terminase_2 (1.9E-40) |
| **2** | 660 | 1925 | 1266 | 421 | 49.4 (6.94) | Terminase large subunit | *S. epidermidis* BCM-HMP0060 (0.0) | 99% (99%) | ZP_04824918.1 | PF04466 Terminase_3 (6.6E-110) |
| **3** | 1931 | 3367 | 1437 | 478 | 56.0 (4.45) | Portal protein | *Staphylococcus* phage CNPH82 (0.0) | 99% (99%) | YP_950601.1 | PF05133 Phage_prot_Gp6 (5.7E-114) |
| **4** | 3324 | 4277 | 954 | 317 | 36.6 (9.30) | Minor head protein | *Staphylococcus* phage CNPH82 (0.0) | 100% (100%) | YP_950602.1 | PF04233 Phage_Mu_F (1.3E-25) |
| **5** | 4385 | 4591 | 207 | 68 | 79.0 (5.16) | Hypothetical protein | *Staphylococcus* phage CNPH82 (3e-29) | 100% (100%) | YP_950604.1 |  |
| **6** | 4706 | 5302 | 597 | 198 | 22.8 (4.54) | Scaffolding protein | *Staphylococcus* phage PH15 (6e-107) | 99% (99%) | YP_950668.1 | PF06810 Phage_GP20 (8.5E-45) |
| **7** | 5320 | 6150 | 831 | 276 | 29.5 (4.89) | Major head protein | *Staphylococcus* phage PH15 (6e-155) | 99% (99%) | YP_950669.1 | PF05065 Phage_capsid (4.4E-7) |
| **8** | 6167 | 6457 | 291 | 96 | 11.2 (4.91) | rho termination factor | *S.*  *epidermidis* M23864:W2 (7e-47) | 99% (100%) | ZP_06614654.1 | PF07498 Rho_N (2.1E-12) |
| **9** | 6457 | 6771 | 315 | 104 | 12.0 (5.67) | DNA packaging protein | *S.*  *epidermidis* BCM-HMP0060 (7e-55) | 99% (100%) | ZP_04824926.1 | PF05135 Phage_QLRG (1.8E-15) |
| **10** | 6764 | 7093 | 330 | 109 | 12.6 (4.97) | Head-tail adaptor | *Staphylococcus* phage PH15 (2e-56) | 98% (99%) | YP_950672.1 | TIGR01563 gp16_SPP1:putative phage head-tail adaptor (6.0E-8) |
| **11** | 7086 | 7499 | 414 | 137 | 15.4 (9.57) | Hypothetical protein | *Staphylococcus* phage PH15 (5e-74) | 99% (99%) | YP_950673.1 | PF04883 DUF646 (6.8E-12) |
| **12** | 7512 | 7949 | 438 | 145 | 16.9 (8.51) | Hypothetical protein | *S.*  *epidermidis* M23864:W2 (6e-79) | 98% (99%) | ZP_06614658.1 |  |
| **13** | 7936 | 8475 | 540 | 179 | 19.9 (4.58) | Major tail protein | *S.*  *epidermidis* BCM-HMP0060 (2e-99) | 99% (100%) | ZP_04824931.1 |  |
| **14** | 8538 | 9032 | 495 | 164 | 18.7 (4.52) | Tail protein | *Staphylococcus* phage CNPH82 (4e-90) | 99% (100%) | YP_950613.1 | PF12363 DUF3647 (1.1E-36) |
| **15** | 9095 | 9397 | 303 | 100 | 11.7 (11.17) | Tail protein | *Staphylococcus* phage PH15 (2e-50) | 100% (100%) | YP_950677.1 |  |
| **16** | 9400 | 12504 | 3105 | 1034 | 111.9 (10.93) | Tail tape measure protein | *S.*  *epidermidis* M23864:W2 (0.0) | 98% (99%) | ZP_06614662.1 |  |
| **17** | 12520 | 13458 | 939 | 312 | 36.3 (6.59) | Tail protein | *Staphylococcus* phage CNPH82 (0.0) | 99% (99%) | YP_950616.1 | PF05709 Sipho_tail (2.8E-32) |
| **18** | 13472 | 15328 | 1857 | 618 | 69.6 (9.12) | Virion associated hydrolase | *S.*  *epidermidis* BCM-HMP0060 (0.0) | 99% (99%) | ZP_04824936.1 | PF00657 Lipase_GDSL (1.6E-9) SSF52266 SGNH hydrolase (1.6E-22) |
| **19** | 15343 | 18009 | 2667 | 888 | 96.8 (6.88) | Pre neck appendage protein | *Staphylococcus* phage CNPH82 (0.0) | 99% (99%) | YP_950618.1 | SSF51126 Pectin lyase-like (1.6E-51) PF11962 DUF3476 (2.4E-83) |
| **20** | 18009 | 19541 | 1533 | 510 | 57.9 (4.69) | Hypothetical protein | *Staphylococcus* phage PH15 (0.0) | 99% (99%) | YP_950682.1 | PF10651 DUF2479 (4.9E-60) |
| **21** | 19546 | 19884 | 339 | 112 | 12.9 (4.19) | Hypothetical protein | *Staphylococcus* phage PH15 (2e-61) | 99% (99%) | YP_950683.1 |  |
| **22** | 19886 | 20026 | 141 | 46 | 5.5 (9.52) | Hypothetical protein | *Staphylococcus* phage PH15 (8e-18) | 100% (100%) | YP_950683.1 | PF09693 Phage_XkdX (4.9E-17) |
| **23** | 20066 | 20473 | 408 | 135 | 14.9 (7.14) | Hypothetical protein | *S.*  *epidermidis* M23864:W2 (2e-61) | 91% (98%) | ZP_06614669.1 |  |
| **24** | 20458 | 20904 | 447 | 148 | 17.0 (5.49) | Exonuclease | *S.*  *epidermidis* M23864:W2 (3e-75) | 96% (99%) | ZP_06614670.1 |  |
| **25** | 21288 | 23111 | 1824 | 607 | 69.8 (10.08) | Peptidoglycan hydrolase | *S.*  *epidermidis* BCM-HMP0060 (0.0) | 91% (95%) | ZP_04824942.1 | PFAM PF01832 Glucosaminidase (4.1E-7) PFAM PF05257 CHAP (5.8E-20) Lysozyme domain, subfamily (2 3.2E-49) |
| **26** | 23164 | 24063 | 900 | 299 | 33.4 (4.55) | Tail fiber protein | *S.*  *epidermidis* BCM-HMP0060 (1e-164) | 91% (91%) | ZP_04824943.1 | PFAM PF10651 DUF2479 (1.5E-35) |
| **27** | 24075 | 24428 | 354 | 117 | 13.5 (4.15) | Hypothetical protein | *Staphylococcus* phage CNPH82 (3e-62) | 100% (100%) | YP_950625.1 |  |
| **28** | 24623 | 24916 | 294 | 97 | 10.6 (7.76) | Holin | *S.*  *epidermidis* BCM-HMP0060 (3e-48) | 100% (100%) | ZP_04824946.1 | PF04531 Phage_holin_1 (1.7E-13) |
| **29** | 24929 | 26653 | 1725 | 574 | 65.8 (9.74) | Lysin | *S.*  *epidermidis* BCM-HMP0060 (0.0) | 100% (100%) | ZP_04824947.1 | PF01510 Amidase_2 (1.0E-10) PF05257 CHAP (1.4E-13) |
| **30** | 28032 | 26983 | 1048 | 349 | 40.7 (9.99) | Integrase | *S.*  *epidermidis* BCM-HMP0060 (0.0) | 100% (100%) | ZP_04824876.1 | PF00589 Phage_integrase (1.1E-20) |
| **31** | 28636 | 28091 | 544 | 181 | 19.8 (8.98) | Hypothetical protein | *S.*  *epidermidis* BCM-HMP0060 (2e-96) | 100% (100%) | ZP_04824877.1 |  |
| **32** | 29114 | 28638 | 475 | 158 | 18.3 (9.59) | Hypothetical protein | *S.*  *epidermidis* BCM-HMP0060 (1e-85) | 100% (100%) | ZP_04824878.1 |  |
| **33** | 29369 | 29202 | 166 | 55 | 6.6 (4.50) | Hypothetical protein | *S.*  *epidermidis* BCM-HMP0060 (3e-23) | 100% (100%) | ZP_04824879.1 |  |
| **34** | 29859 | 29494 | 364 | 121 | 13.9 (4.79) | Repressor protein | *S.*  *epidermidis* BCM-HMP0060 (8e-65) | 100% (100%) | ZP_04824880.1 | PF00717 Peptidase_S24 (1.1E-8) |
| **34*** | 30168 | 29941 | 226 | 75 | 8.6 (9.99) | Repressor protein |  |  |  |  |
| **35** | 30384 | 30596 | 213 | 70 | 8.2 (7.17) | Cro | *S.*  *epidermidis* BCM-HMP0060 (4e-32) | 100% (100%) | ZP_04824881.1 |  |
| **36** | 30610 | 30771 | 162 | 53 | 6.2 (5.51) | Hypothetical protein | *S.*  *epidermidis* BCM-HMP0060 (4e-20) | 100% (100%) | ZP_04824882.1 |  |
| **37** | 31065 | 31820 | 756 | 251 | 28.8 (6.74) | Antirepressor protein | *S.*  *epidermidis* BCM-HMP0060 (9e-144) | 100% (100%) | ZP_04824884.1 | PF03374 ANT (1.0E-34) |
| **38** | 31833 | 32027 | 195 | 64 | 7.5 (10.38) | Hypothetical protein | *Staphylococcus* *capitis* SK14 (1e-05) | 65% (77%) | ZP_03614384.1 |  |
| **39** | 32243 | 32452 | 210 | 69 | 8.1 (8.62) | Hypothetical protein | *S.*  *epidermidis* BCM-HMP0060 (6e-33) | 100% (100%) | ZP_04824886.1 |  |
| **40** | 32516 | 32692 | 177 | 58 | 6.7 (9.38) | Hypothetical protein | *S.*  *epidermidis* BCM-HMP0060 (6e-24) | 100% (100%) | ZP_04824887.1 |  |
| **41** | 32747 | 33022 | 276 | 91 | 10.8 (4.99) | Hypothetical protein | *S.*  *epidermidis* BCM-HMP0060 (2e-45) | 100% (100%) | ZP_04824888.1 | PF02346 Vac_Fusion (0.0017) |
| **42** | 33211 | 33855 | 645 | 214 | 24.5 (6.65) | Recombination protein | *S.*  *epidermidis* BCM-HMP0060 (2e-122) | 100% (100%) | ZP_04824890.1 | PF04404 ERF (6.3E-38) |
| **43** | 33855 | 34265 | 411 | 136 | 15.0 (8.69) | Single strand DNA binding protein | *Staphylococcus* phage CNPH82 (6e-73) | 100% (100%) | YP_950641.1 | PF00436 SSB (2.0E-23) |
| **44** | 34279 | 34953 | 675 | 224 | 26.1 (6.49) | Hypothetical protein | *S.*  *epidermidis* BCM-HMP0060 (8e-130) | 100% (100%) | ZP_04824892.1 | PF06147 DUF968 (1.7E-65) |
| **45** | 34950 | 35651 | 702 | 233 | 26.9 (8.69) | DNA replication protein | *S.*  *epidermidis* BCM-HMP0060 (2e-132) | 99% (100%) | ZP_04824893.1 | PF07261 DnaB_2 (3.7E-12) |
| **46** | 35657 | 36010 | 354 | 117 | 14.5 (9.23) | Hypothetical protein | *Staphylococcus* phage CNPH82 (4e-62) | 100% (100%) | YP_950644.1 |  |
| **47** | 35997 | 37247 | 1251 | 416 | 47.5 (4.74) | DNA helicase | *S.*  *epidermidis* BCM-HMP0060 (0.0) | 100% (100%) | ZP_04824895.1 | PF03796 DnaB_C (3.6E-47) |
| **48** | 37244 | 37465 | 222 | 73 | 8.8 (5.71) | Hypothetical protein | *S.*  *epidermidis* BCM-HMP0060 (1e-35) | 100% (100%) | ZP_04824896.1 |  |
| **49** | 37443 | 37688 | 246 | 81 | 9.5 (9.45) | Hypothetical protein | *S.*  *epidermidis* BCM-HMP0060 (2e-40) | 100% (100%) | ZP_04824897.1 | PF11673 DUF3269 (3.4E-6) |
| **50** | 37697 | 38104 | 408 | 135 | 15.9 (9.66) | Resolvase | *S.*  *epidermidis* BCM-HMP0060 (3e-74) | 100% (100%) | ZP_04824898.1 | PF05866 RusA (1.09E-26) |
| **51** | 38105 | 38302 | 198 | 65 | 7.6 (4.33) | Transcriptional regulator | *S.*  *epidermidis* BCM-HMP0060 (5e-29) | 100% (100%) | ZP_04824899.1 |  |
| **52** | 38303 | 38662 | 360 | 119 | 14.2 (9.96) | Hypothetical protein | *S.*  *epidermidis* BCM-HMP0060 (3e-63) | 100% (100%) | ZP_04824900.1 | PF07768 PVL_ORF50 (1.2E-36) |
| **53** | 38659 | 39114 | 456 | 151 | 17.5 (4.62) | Hypothetical protein | *S.*  *epidermidis* BCM-HMP0060 (2e-82) | 100% (100%) | ZP_04824901.1 | PF11753 DUF3310 (4.19E-22) |
| **54** | 39119 | 39349 | 231 | 76 | 8.5 (4.69) | Hypothetical protein | *S.*  *epidermidis* BCM-HMP0060 (7e-35) | 100% (100%) | ZP_04824902.1 |  |
| **55** | 39355 | 39969 | 615 | 204 | 23.6 (10.44) | Hypothetical protein | *S.*  *epidermidis* BCM-HMP0060 (3e-116) | 100% (100%) | ZP_04824903.1 |  |
| **56** | 39956 | 40153 | 198 | 65 | 7.4 (10.38) | Hypothetical protein | *S.*  *epidermidis* BCM-HMP0060 (4e-30) | 100% (100%) | ZP_04824904.1 |  |
| **57** | 40140 | 40319 | 180 | 59 | 6.8 (4.43) | Hypothetical protein | *S.*  *epidermidis* BCM-HMP0060 (4e-26) | 100% (100%) | ZP_04824905.1 |  |
| **58** | 40336 | 40536 | 201 | 66 | 7.8 (4.52) | Hypothetical protein | *S.*  *epidermidis* BCM-HMP0060 (6e-31) | 100% (100%) | ZP_04824906.1 |  |
| **59** | 40562 | 40951 | 390 | 129 | 14.9 (4.07) | Yopx protein | *S.*  *epidermidis* BCM-HMP0060 (6e-69) | 100% (100%) | ZP_04824907.1 | PF09643 YopX (1.79E-25) |
| **60** | 40952 | 41296 | 345 | 114 | 13.1 (4.96) | Nuclease | *S.*  *epidermidis* BCM-HMP0060 (2e-59) | 99% (100%) | ZP_04824908.1 | PF00565 SNase (3.0E-10) |
| **61** | 41301 | 41453 | 153 | 50 | 5.8 (10.17) | Hypothetical protein | *S.*  *epidermidis* BCM-HMP0060 (3e-19) | 100% (100%) | ZP_04824909.1 |  |
| **62** | 41443 | 41622 | 180 | 59 | 6.7 (6.08) | Hypothetical protein | *S.*  *epidermidis* BCM-HMP0060 (7e-27) | 100% (100%) | ZP_04824910.1 | PF06260 DUF1024 (2.4E-8) |
| **63** | 41615 | 42157 | 543 | 180 | 19.8 (4.29) | dUTP diphosphatase | *S.*  *epidermidis* BCM-HMP0060 (5e-99) | 100% (100%) | ZP_04824911.1 | PF00692 dUTPase (5.89E-6) |
| **64** | 42194 | 42406 | 213 | 70 | 8.2 (9.49) | Hypothetical protein | *S.*  *epidermidis* BCM-HMP0060 (4e-32) | 99% (100%) | ZP_04824912.1 | PF07129 DUF1381 (4.7E-24) |
| **65** | 42403 | 42582 | 180 | 59 | 7.0 (6.33) | rinB | *S.*  *epidermidis* BCM-HMP0060 (5e-26) | 99% (100%) | ZP_04824913.1 | PF06116 RinB (1.3E-32) |
| **66** | 42722 | 42940 | 219 | 72 | 8.5 (9.81) | Hypothetical protein | *Staphylococcus* phage PH15 (4e-33) | 99% (100%) | YP_950729.1 |  |
| **67** | 42958 | 43374 | 417 | 138 | 16.4 (5.40) | rinA | *Staphylococcus* phage PH15 (9e-74) | 99% (100%) | YP_950730.1 | TIGR01636 phage_rinA: phage transcriptional regulator (1.1E-10) |
