# Supplementary material for: Genomic characterization of two Staphylococcus epidermidis bacteriophages with anti-biofilm potential
Source: BMC Genomics. 2012 Jun 8;13:228. doi: 10.1186/1471-2164-13-228 (PMC3505474; doi:10.1186/1471-2164-13-228)
Supplement: Additional file 2 — Table S2. Features of bacteriophage phi-IPLA7 orfs, gene products (gp) and functional assignments. [file 1471-2164-13-228-S2.doc]

**Table 2.** Features of bacteriophage phi-IPLA7 *orf*s, gene products (gp) and functional assignments.

| ***orf*** | **From** | **To** | **Length** | **aa** | **kDa (pI)** | **Predictive Function** | **Closes hit (E value)** | **% aa identity / % similarity** | **Accesion no.** | **Predicted domain (E value)** |
| --- | --- | --- | --- | --- | --- | --- | --- | --- | --- | --- |
| **1** | 239 | 619 | 381 | 126 | 14.5 (5.52) | Terminase small subunit | *S. epidermidis* BCM-HMP0060 (5e-69) | 100% (100%) | ZP_04824917.1 | PF04297 |
| **2** | 606 | 1868 | 1263 | 420 | 49.2 (7.73) | Terminase large subunit | *S. epidermidis* BCM-HMP0060 (0.0) | 100% (100%) | ZP_04824918.1 | PF04466 Terminase_3 (2.9E-107) |
| **3** | 1874 | 3310 | 1437 | 478 | 56.0 (4.50) | Portal protein | *Staphylococcus* phage CNPH82 (0.0) | 99% (99%) | YP_950601.1 | PF05133 Phage_prot_Gp6 (7.3E-113) |
| **4** | 3267 | 4214 | 948 | 315 | 36.5 (9.19) | Minor head protein | *Staphylococcus* phage CNPH82 (0.0) | 98% (99%) | YP_950602.1 | PF04233 Phage_Mu_F (2.6E-28) |
| **5** | 4308 | 4901 | 594 | 197 | 22.2 (4.37) | Minor head protein | *S. epidermidis* M23864:W2 (5e-105) | 98% (99%) | ZP_06614652.1 | PF06810 Phage_GP20 (8.7E-48) |
| **6** | 4919 | 5749 | 831 | 276 | 29.5 (4.99) | Major head protein | *S. epidermidis* M23864:W2 (2e-155) | 99% (100%) | ZP_06614653.1 | PF05065 Phage_capsid (1.3E-6) |
| **7** | 5766 | 6056 | 291 | 96 | 11.2 (4.66) | rho termination factor | *S. epidermidis* M23864:W2 (2e-47) | 100% (100%) | ZP_06614654.1 | PF07498 Rho_N (6.8E-10) |
| **8** | 6056 | 6370 | 315 | 104 | 12.0 (7.02) | Head protein | *Staphylococcus* phage CNPH82 (4e-55) | 100% (100%) | YP_950608.1 | PF05135 Phage_QLRG (8.2E-16) |
| **9** | 6363 | 6692 | 330 | 109 | 12.6 (4.96) | Head-tail protein | *Staphylococcus* phage PH15 (1e-57) | 100% (100%) | YP_950672.1 | TIGR01563 gp16_SPP1: putative phage head-tail adaptor (3.8E-7) |
| **10** | 6685 | 7098 | 414 | 137 | 15.4 (9.42) | Hypothetical protein | *Staphylococcus* phage PH15 (2e-74) | 99% (99%) | YP_950673.1 | PF04883 DUF646 (1.6E-19) |
| **11** | 7111 | 7548 | 438 | 145 | 16.9 (8.51) | Hypothetical protein | *S. epidermidis* M23864:W2 (7e-78) | 96% (98%) | ZP_06614658.1 |  |
| **12** | 7535 | 8074 | 540 | 179 | 19.9 (4.54) | Major tail protein | *Staphylococcus* phage CNPH82 (1e-99) | 100% (100%) | YP_950612.1 |  |
| **13** | 8136 | 8630 | 495 | 164 | 18.7 (4.57) | Hypothetical protein | *S. epidermidis* BCM-HMP0060 (3e-90) | 99% (99%) | ZP_04824932.1 | PF12363 DUF3647 (5.2E-36) |
| **14** | 8693 | 8995 | 303 | 100 | 11.7 (11.17) | Hypothetical protein | *Staphylococcus* phage PH15 (2e-50) | 100% (100%) | YP_950677.1 |  |
| **15** | 8998 | 12102 | 3105 | 1034 | 112.1 (10.91) | Tail tape measure protein | *S. epidermidis* M23864:W2 (0.0) | 99% (99%) | ZP_06614662.1 |  |
| **16** | 12118 | 13056 | 939 | 312 | 36.4 (6.10) | Hypothetical protein | *Staphylococcus* phage CNPH82 (7e-177) | 97% (99%) | YP_950616.1 | PF05709 Sipho_tail (1.3E-41) |
| **17** | 13070 | 14926 | 1857 | 618 | 69.6 (9.11) | Hydrolase | *S. epidermidis* BCM-HMP0060 (0.0) | 99% (99%) | ZP_04824936.1 | PF00657 Lipase_GDSL (2.8E-9) SSF52266 SGNH hydrolase (1.0E-22) |
| **18** | 14941 | 17607 | 2667 | 888 | 96.8 (6.65) | Pre neck appendage protein | *Staphylococcus* phage CNPH82 (0.0) | 99% (99%) | YP_950618.1 | SSF51126 Pectin lyase-like (1.1E-51) |
| **19** | 17607 | 19139 | 1533 | 510 | 57.9 (4.74) | Hypothetical protein | *Staphylococcus* phage CNPH82 (0.0) | 100% (100%) | YP_950619.1 | PF10651 DUF2479 (6.5E-57) |
| **20** | 19123 | 19482 | 360 | 119 | 13.6 (4.10) | Hypothetical protein | *Staphylococcus* phage CNPH82 (3e-62) | 100% (100%) | YP_950620.1 |  |
| **21** | 19628 | 19960 | 333 | 110 | 12.8 (7.62) | Hypothetical protein | *Staphylococcus* phage CNPH82 (8e-56) | 99% (100%) | YP_950622.1 | PF11166 DUF2951 (5.4E-48) |
| **22** | 20099 | 22000 | 1902 | 633 | 72.4 (10.11) | Amidase | *Staphylococcus* phage CNPH82 (0.0) | 99% (99%) | YP_950623.1 | PF01832 Glucosaminidase (2.4E-7) PF05257 CHAP (1.2E-25) |
| **23** | 22054 | 22563 | 510 | 169 | 19.2 (4.28) | Hypothetical protein | *Staphylococcus* phage CNPH82 (3e-91) | 98% (99%) | YP_950687.1 |  |
| **24** | 22563 | 23090 | 528 | 175 | 20.4 (4.51) | Hypothetical protein | *S. epidermidis* M23864:W2 (9e-82) | 88% (93%) | ZP_06614676.1 |  |
| **25** | 23140 | 23412 | 273 | 90 | 9.8 (9.89) | Holin | *Staphylococcus* phage CNPH82 (7e-45) | 100% (100%) | YP_950627.1 | PF04688 Phage_holin (1.09E-16) |
| **26** | 23412 | 24794 | 1383 | 460 | 52.5 (9.75) | Lysin | *Staphylococcus* phage CNPH82 (0.0) | 99% (100%) | YP_950628.1 | PF01520 Amidase_3 (2.1E-26) PF05257 CHAP (6.5E-21) |
| **27** | 24872 | 25798 | 927 | 308 | 36.4 (9.53) | Hypothetical protein | *Staphylococcus* phage PH15 (1e-171) | 100% (100%) | YP_950691.1 |  |
| **28** | 27458 | 26082 | 1375 | 458 | 54.2 (9.12) | Integrase | *Staphylococcus* phage CNPH82 (0.0) | 100% (100%) | YP_950630.1 | PF00239 Resolvase (1.6E-37) PF07508 Recombinase (3.1E-16) |
| **29** | 28325 | 27510 | 814 | 271 | 29.8 (4.87) | Hypothetical protein | *S. epidermidis* M23864:W2 (3e-144) | 98% (99%) | ZP_06614616.1 |  |
| **30** | 28771 | 28322 | 448 | 149 | 17.6 (5.81) | Hypothetical protein | *Staphylococcus* phage EW (1e-35) | 49% (73%) | YP_240186.1 | PF06114 DUF955 (9.6E-15) |
| **31** | 29120 | 28806 | 313 | 104 | 12.1 (5.80) | Repressor | *Staphylococcus carnosus* subsp. *carnosus* TM300 (6e-29) | 60% (80%) | YP_002633565.1 | PF01381 HTH_3 (4.4E-15) |
| **32** | 29286 | 29531 | 246 | 81 | 9.3 (10.14) | Cro | *Staphylococcus* phage 2638A (4e-13) | 50% (79%) | YP_239824.1 | PF01381 HTH_3 (7.4E-6) |
| **33** | 29544 | 30296 | 753 | 250 | 28.7 (9.48) | Antirepressor | *Staphylococcus* phage phiPVL108 (3e-135) | 93% (97%) | YP_918898.1 | PF03374 ANT (3.9E-39) PF08346 AntA (8.8E-24) |
| **34** | 30310 | 30477 | 168 | 55 | 6.1 (3.77) | Hypothetical protein | *Staphylococcus* *caprae* C87 (1e-19) | 99% (100%) | [ZP_07841352.1|](http://www.ncbi.nlm.nih.gov/protein/314933987?report=genbank&log$=protalign&blast_rank=1&RID=PRFZKS2A01S) |  |
| **35** | 30571 | 30747 | 177 | 58 | 6.6 (8.75) | Hypothetical protein | *S. epidermidis* M23864:W2 (2e-22) | 95% (99%) | ZP_06614621.1 |  |
| **36** | 30802 | 31077 | 276 | 91 | 10.9 (4.63) | Hypothetical protein | *Staphylococcus* phage PH15 (3e-44) | 97% (100%) | YP_950706.1 |  |
| **37** | 31269 | 31940 | 672 | 223 | 25.7 (5.98) | erf protein | *Staphylococcus* *lugdunensis* HKU09-01 (2e-116) | 92% (97%) | YP_003472492.1 | PF04404 ERF (1.1E-36) |
| **38** | 31940 | 32362 | 423 | 140 | 15.5 (5.23) | Single strand DNA binding protein | *S. epidermidis* M23864:W2 (4e-75) | 100% (100%) | ZP_06612891.1 | PF00436 SSB (1.79E-20) |
| **39** | 32376 | 33050 | 675 | 224 | 26.2 (7.31) | Hypothetical protein | *Staphylococcus* phage PH15 (1e-125) | 96% (99%) | YP_950710.1 | PF06147 DUF968 (8.7E-68) |
| **40** | 33043 | 33843 | 801 | 266 | 31.1 (5.64) | DNA replication protein | *Staphylococcus* phage PH15 (8e-115) | 82% (93%) | YP_950711.1 | PF07261 DnaB_2 (2.6E-11) PF09681 Phage_rep_org_N (6.2E-45) |
| **41** | 33843 | 34202 | 360 | 119 | 14.1 (9.31) | Hypothetical protein | *Staphylococcus* phage PH15 (2e-63) | 100% (100%) | YP_950712.1 |  |
| **42** | 34195 | 35430 | 1236 | 411 | 47.1 (5.12) | DNA helicase | *Staphylococcus* phage PH15 (0.0) | 99% (100%) | YP_950713.1 | PF03796 DnaB_C (1.4E-45) |
| **43** | 35427 | 35648 | 222 | 73 | 8.7 (7.13) | Hypothetical protein | *Staphylococcus* phage PH15 (3e-35) | 100% (100%) | YP_950714.1 |  |
| **44** | 35626 | 35871 | 246 | 81 | 9.5 (9.51) | Hypothetical protein | *S. epidermidis* M23864:W2 (3e-40) | 100% (100%) | ZP_06614632.1 | PF11673 DUF3269 (0.0085) |
| **45** | 35880 | 36287 | 408 | 135 | 15.9 (9.66) | Resolvase | *Staphylococcus* phage PH15 (5e-74) | 100% (100%) | YP_950716.1 | PF05866 RusA (3.5E-26) |
| **46** | 36288 | 36479 | 192 | 63 | 7.4 (4.08) | Hypothetical protein | *S. epidermidis* M23864:W2(3e-27) | 99% (100%) | ZP_06612901.1 |  |
| **47** | 36480 | 36893 | 414 | 137 | 16.8 (9.42) | Hypothetical protein | *S. epidermidis* M23864:W2 (4e-12) | 72% (83%) | ZP_06614635.1 | PF07768 PVL_ORF50 (8.1E-19) |
| **48** | 36890 | 37333 | 444 | 147 | 17.1 (9.14) | Hypothetical protein | *S. epidermidis* M23864:W2 (4e-63) | 79% (89%) | ZP_06614636.1 | PF11753 DUF3310 (1.4E-25) |
| **49** | 37336 | 38016 | 681 | 226 | 26.6 (9.53) | Endonuclease | *Staphylococcus* phage CNPH82 (1e-126) | 97% (99%) | YP_950653.1 |  |
| **50** | 38064 | 38243 | 180 | 59 | 6.8 (4.67) | Hypothetical protein | *S. epidermidis* BCM-HMP0060 (7e-24) | 94% (97%) | ZP_04824905.1 |  |
| **51** | 38262 | 38648 | 387 | 128 | 14.8 (4.63) | Hypothetical protein | *Staphylococcus* *haemolyticus* JCSC1435 (5e-35) | 54% (75%) | YP_254286.1 |  |
| **52** | 38652 | 38939 | 288 | 95 | 11.2 (4.78) | Hypothetical protein | *Staphylococcus* phage PH15 (1e-33) | 95% (95%) | YP_950721.1 | PF05405 Mt_ATP-synt_B (1.3E-4) |
| **53** | 38929 | 39177 | 249 | 82 | 9.8 (5.46) | Hypothetical protein | *Staphylococcus* phage PH15 (9e-41) | 100% (100%) | YP_950722.1 |  |
| **54** | 39333 | 39569 | 237 | 78 | 9.0 (4.65) | Hypothetical protein |  |  |  |  |
| **55** | 39717 | 40040 | 324 | 107 | 12.0 (4.76) | DNA binding protein | *S. haemolyticus* JCSC1435 (1e-47) | 91% (96%) | YP_253703.1 |  |
| **56** | 40538 | 40708 | 171 | 56 | 6.6 (5.81) | rinB | *S. epidermidis* M23864:W2 (7e-23) | 90% (100%) | ZP_06614643.1 | PF06116 RinB (2.7E-31) |
| **57** | 40686 | 41129 | 444 | 147 | 16.8 (8.20) | Hypothetical protein | *S. epidermidis* M23864:W2 (1e-39) | 63% (72%) | ZP_06614644.1 |  |
| **58** | 41261 | 41479 | 219 | 72 | 8.5 (9.78) | Hypothetical protein | *Staphylococcus* phage PH15 (2e-33) | 100% (100%) | YP_950729.1 |  |
| **59** | 41497 | 41913 | 417 | 138 | 16.4 (5.40) | rinA | *Staphylococcus* phage PH15 (4e-74) | 100% (100%) | YP_950730.1 | TIGR01636 phage_rinA: phage transcriptional regulator (5.1E-11) |
