# Supplementary material for: Genomic characterization of two Staphylococcus epidermidis bacteriophages with anti-biofilm potential
Source: BMC Genomics. 2012 Jun 8;13:228. doi: 10.1186/1471-2164-13-228 (PMC3505474; doi:10.1186/1471-2164-13-228)
Supplement: Additional file 3 — Table S3. Putative promoters and terminators sequences of phi-IPLA5 and phi-IPLA7. -10 and -35 boxes are underlined. Nucleotide positions and presence of the TG dinucleotide were also indicated. At terminator sequences nucleotides in the stem-loop structure are underlined. [file 1471-2164-13-228-S3.doc]

**Table S1.** Putative promoters and terminators sequences of phi-IPLA5 and phi-IPLA7. -10 and -35 boxes are underlined. Nucleotide positions and presence of the TG dinucleotide were also indicated. At terminator sequences nucleotides in the stem-loop structure are underlined.

| Promoter phi-IPLA5 | Putative promoter sequence (5’-3’) | Nt position | Spacer | TG | Prior to gene |
| --- | --- | --- | --- | --- | --- |
| P1 | TTTAATAAGTATTTTTCATAAGGTGGTTAAGAT | 7078-7110 | 18 | - | *orf11* |
| P2 | TCGAGTCAACGCAATGCGTTGGCTTTTAATTT | 23093-23125 | 17 | - | *orf25* |
| P3 | TTGTAAATAAACAACACGTAGTGTATATT | 30206-30236 | 15 | + | *orf34* |
| P4 | TTGCTAAAGACTACCAATTCATGTATTAT | 30286-30314 | 15 | + | *orf35* |
| P5 | TTGAAAGAAACAGTGACATATCTAATTAAACT | 31049-31080 | 17 | - | *orf37* |
| P6 | TTTATTTGCTATCAATTAATTAAAAT | 39279-39304 | 11 | - | *orf54* |
| P7 | TTGTAGATAGTATTACTGCTGATACGAT | 40244-40271 | 13 | + | *orf56* |
| P8 | TTGAAGTGTGTAAGTCCGTTTATAAT | 41923-41948 | 11 | - | *orf1* |
|  |  |  |  |  |  |
| Terminator  phi-IPLA5 | Putative terminator sequence (5’-3’) | Nt position | Stem lenght | ΔG kcal/mol | After to gene |
| T1 | GAGGGCAUUAAGCCCUCUAUUUUUUU | 8082-8107 | 6 | -11.40 | *orf12* |
| T2 | GGCUACUCACUGACGGUGGGUAGCCUUU | 22004-22031 | 10 | -17.40 | *orf22* |
| T3 | GCGGGGAAACCCGCUUUUUUU | 24804-24824 | 5 | -11.80 | *orf26* |
| T4 | CCACGUUCUUUAUGAGCGUGGUUAUUUUU | 26000-26028 | 8 | -11.10 | *orf27* |
|  |  |  |  |  |  |
| Promoter phi-IPLA7 | Putative promoter sequence (5’-3’) | Nt position | Spacer | TG | Prior to gene |
| P1 | TTTCAATAAGTATTTTTCATAAGGTGGTTAAGAT | 7478-7511 | 19 | - | *orf12* |
| P2 | GTCAATACATAGGCTATCTTCCTTATGAT | 9060-9088 | 14 | - | *orf15* |
| P3 | TTGACAGCCGACGTTTTGCGTTGGCTTTTATTTT | 24573-24607 | 19 | - | *orf28* |
| P4 | TTGTACATTTCCGGAAATTGTGTATAAT | 29152-29179 | 14 | + | *orf31* |
| P5 | TTGACTTCGGAATTACCGAAATGTTATCAT | 29224-29253 | 16 | - | *orf32* |
| P6 | TTGCCAATCAACGTGGATTGTTACGAT | 30858-30883 | 12 | + | *orf37* |
| P7 | TTAAATATACAAGTGGAGGAGAGAAAAT | 32721-32748 | 14 | - | *orf41* |
| P8 | GTGTAAGTCCGTTTATAACCAATGTATTAT | 43391-43420 | 16 | + | *orf1* |
|  |  |  |  |  |  |
| Terminator  phi-IPLA7 | Putative terminator sequence (5’-3’) | Nt position | Stem lenght | ΔG kcal/mol | After to gene |
| T1 | GCUAACACUAAAAUGUGUUGGCUAUUUUUU | 4591-4620 | 8 | -10.70 | *orf5* |
| T2 | GAGGGCAUUAAGCCCUCUAUUUUUUU | 8483-8508 | 6 | -11.40 | *orf13* |
| T3 | CCGCAAGUUAAAUAACUUGUGGUUUUU | 20012-20038 | 9 | -10.20 | *orf22* |
| T4 | GGCUACUCACUGACGGUGGGUAGCCUUU | 23114-23141 | 10 | -17.40 | *orf25* |
| T5 | GGGUAGUCACUAUGACUACCCUCUUUUUUU | 26692-26721 | 9 | -14.00 | *orf29* |
