# Supplementary material for: Genomic characterization of two Staphylococcus epidermidis bacteriophages with anti-biofilm potential
Source: BMC Genomics. 2012 Jun 8;13:228. doi: 10.1186/1471-2164-13-228 (PMC3505474; doi:10.1186/1471-2164-13-228)
Supplement: Additional file 4 — Table S4. Primers used for multiplex PCR reactions. [file 1471-2164-13-228-S4.doc]

| **Gene** | **Primer** | **Primer sequence (5’- 3’)** | **Amplicon size (bp)** |
| --- | --- | --- | --- |
| phi-IPLA7 major head protein | MHPphi7-1 | 5’-AGTACATTAGTAGGACAACCAGGTG-3’ | 620 bp |
|  | MHPphi7-2 | 5’- AGTTGTTTTAGTTGAAGGATCACGG-3’ |  |
| phi-IPLA7 integrase | intphi7-1 | 5’-TTTATCTAAGTCTAGTTTAGATAGG-3’ | 470 pb |
|  | intphi7-2 | 5’-GACGAAGTGATGAAAGGTAATAG-3’ |  |
| phi-IPLA5 integrase | intphi5-1 | 5’-GTATGACTAACTGAGAATAGCTTG-3’ | 660 pb |
|  | intphi5-2 | 5’-TAGAAGATGGCAACAACATTGAAG-3’ |  |
| phi-IPLA7 holin | holphi7-1 | 5’- AACAGATGTAGGTTCAATTGTAAG-3’ | 300 pb |
|  | holphi5-2 | 5’- TAGGCCTCCTATTCTTTATTAGG-3’ |  |

**Table S2.** Primers used for multiplex PCR reactions.
