# Supplementary figures and images for: Genomic characterization of two Staphylococcus epidermidis bacteriophages with anti-biofilm potential
Source: BMC Genomics. 2012 Jun 8;13:228. doi: 10.1186/1471-2164-13-228 (PMC3505474; doi:10.1186/1471-2164-13-228)

Figure S1

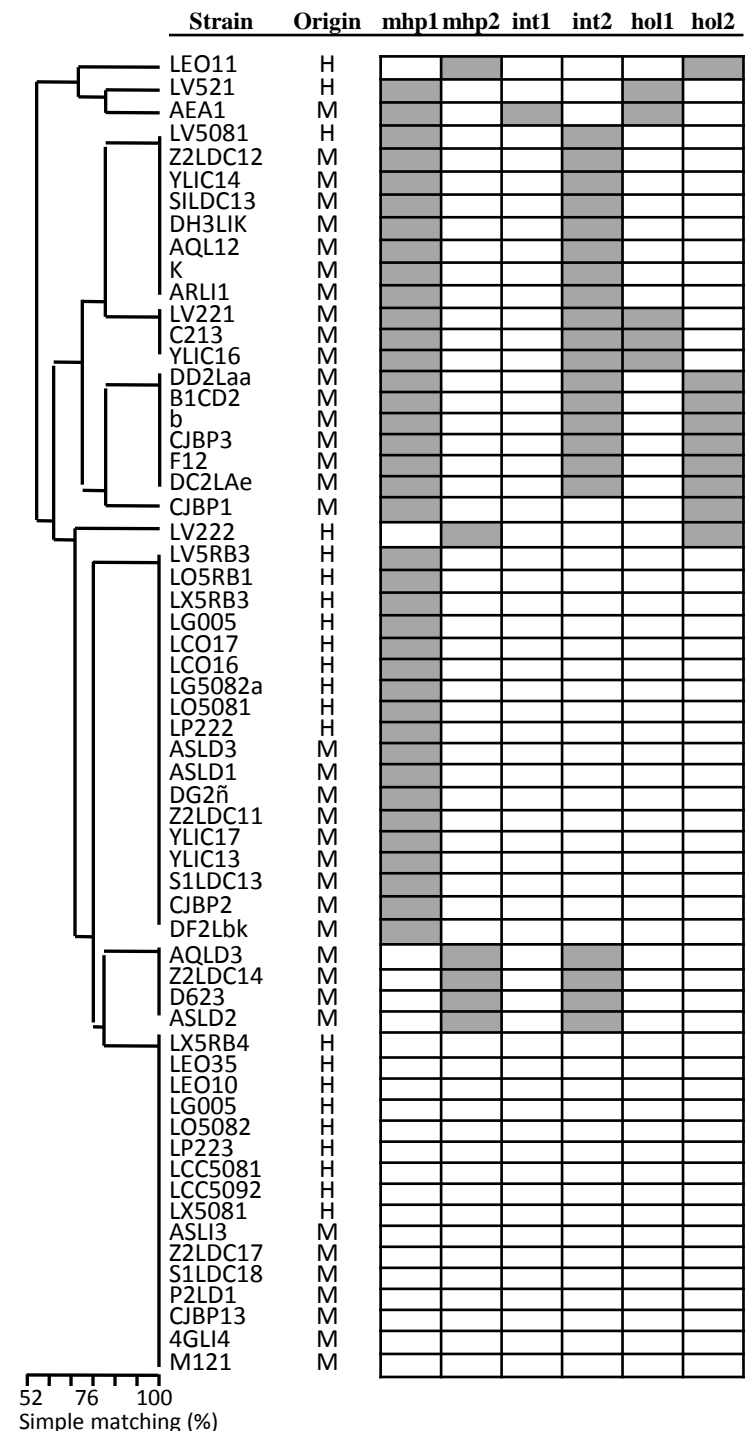

Supplement: Additional file 5 — Figure S1. Multiplex PCR detecting the integrase, holin and major head protein genes in S. epidermidis strains. (H) Strains isolated from healthy woman, (M) strains isolated from mastitic women. Absence and presence of a specific gene is represented by white and grey boxes, respectively. [file 1471-2164-13-228-S5.pdf]
